# Interaction of Shear and Rayleigh–Lamb Waves with Notches and Voids in Plate Waveguides

**DOI:** 10.3390/ma10070841

**Published:** 2017-07-21

**Authors:** Annamaria Pau, Dimitra V. Achillopoulou

**Affiliations:** Department of Structural and Geotechnical Engineering, Sapienza University of Rome, 00185 Rome, Italy; dimitra.achillopoulou@uniroma1.it

**Keywords:** guided waves, damage characterization, scattered fields, reflection and transmission coefficients

## Abstract

This paper investigates the interaction of different shear- and Rayleigh–Lamb-guided waves in plates with a discontinuity such as a notch or an internal void. The problem was solved numerically using a finite element model and by exploiting an analytical solution obtainable for the double sharp changes of the cross-section that served as a reference. We aimed to elucidate the relation between the size and shape of the discontinuity and the reflection and transmission coefficients of the scattered field. Different sizes and profiles of the discontinuity were considered, with the shapes ranging from step changes of the height to ellipses, both symmetric and nonsymmetric. Regimes related to low and high values of the product frequency multiplied by the height of the plate were investigated. These showed how the mode conversion was related to the symmetry between the incident mode and the discontinuity, and to the actual existence of multiple propagating modes. The analysis presented was motivated by the need to set up procedures that exploit propagating waves not only to detect the presence of a notch, but also to characterize its size and shape.

## 1. Introduction

Guided waves play an important role in nondestructive health monitoring, with applications ranging from the detection of cracks and corrosion to the monitoring of states of stress [1,2]. Their success is related to the geometric waveguide structure of many structural elements, such as beams, rails, plates and pipes. With remarkable advantages compared to bulk waves for inspection areas, guided waves propagating in such solids can be used to monitor large structural portions, due to the existence of modes with minimal attenuation. This technique has mainly been used for defect screening rather than defect characterization because of the many difficulties that arise when the scattered field that originates from a wave encountering a discontinuity has to be interpreted. Such an aspect is crucial, as has also been noted by recent reviews of research on guided-wave-based structural health monitoring [3,4]. Moreover, a detailed knowledge of the displacement field can be used to improve the focus in defect image reconstruction when using wave mode beamforming and compounding strategies [5].

Practical guided-wave sonic and ultrasonic testing is performed by sending a signal along a waveguide and interpreting the scattered response. In the simplest case, one single-mode signal is used. A high frequency of excitation is used when it is necessary to detect minute damage, with a size comparable to the magnitude of the excitation wavelength. In the presence of a defect, if the frequency of excitation for some waves is higher than the cutoff frequency, the transmitted and reflected responses can consist of a complex superposition of wave modes, depending on the number of propagating modes that exist in the range of frequencies excited, and on the geometric symmetry of both the wave and the discontinuity. The scattered signal can be a complex multimodal signal, due to mode conversion that occurs as a result of the requirement that boundary conditions are satisfied on the surface of the discontinuity. The dispersive character of many modes further complicates the interpretation, as the shape of the signal changes in time and space with distance [6,7].

The use of guided waves in detecting the presence of and locating a defect is well established, although their application in geometrically sizing and mapping the in-depth profile of the defect is still out of reach. Detailed understanding of wave interactions with defects can be of use in the model-based definition of a procedure for defect characterization based on the scattering response [8]. In fact, the ability to describe the variation of scattering coefficients as a function of the geometric characteristics of the discontinuity is fundamental to define a strategy to solve the inverse problem, and it can also help to select modes and frequencies that improve the inspection sensitivity to various discontinuities. Some applications of guided waves to defect sizing and shape reconstruction of surfaces and inner defects have recently appeared [9,10,11,12,13].

The focus of this paper is the interaction between shear (SH0) and Rayleigh–Lamb (S0) waves with voids and discontinuities of different kinds, in order to elucidate the relationship between the discontinuity size and the profile, and the scattered field. Some aspects of this topic have already been investigated by several researchers in the last two decades. Some of the most important research is listed below.

The interaction of the SH0 mode with discontinuities of various profiles was investigated by Demma et al. [14], although they limited their analysis to low frequencies and were not able to observe mode conversion. In other studies, such as those of Rajagopal and Lowe [6] or Ratassep et al. [7], the diffraction of the SH0 mode was again studied using a finite element model, but only for through-thickness defects. From a practical point of view, the SH0 mode is nondispersive and can be applied to both plates and pipes because its dynamics also satisfactorily describe the behavior of the first torsional mode in pipes of large radii. Its sizing ability was experimentally demonstrated in [15]. The work by Alleyne and Cawley [16] is an important reference on the interaction of Lamb waves with defects. Using finite element models, they determined the scattered field originated by symmetric (S0) and asymmetric (A0) waves encountering notch-type nonsymmetric defects, considering several frequency bands and notch sizes. Various phenomena of mode conversion were observed by Cho [17], and recently, by Schaal and Mal [18], who investigated the interaction of Lamb waves with step discontinuities, and by Wang et al. [19], who dealt with the scattering from internal slot defects.

In the authors’ opinion, the influence of the discontinuity profile on the scattered field deserves investigation. To do so, we chose different profiles, a double sharp discontinuity and an elliptic profile, that were placed inside or on the surface of the waveguide, either symmetrically or nonsymmetrically; several sizes of the discontinuity were considered. The response was calculated with finite element models, although we also made use of an analytical model that was fit to describe the simplest geometrical case—the sharp change in height. This analytical model was based on an integral formulation of the boundary conditions that exploits the principle of reciprocity in elastodynamics [20,21] and is described in detail in [22,23] or, similarly, in [24]. The analytical model improves the understanding of the finite element (FE) results and unveils the background for which the mechanical phenomena of scattering occur. Hence, a short analytical description of guided waves and their interaction with defects is first presented. The simulations presented here provide a valuable tool for research in non-destructive evaluation. They enabled us to define the most sensitive kinds of waves to be used to interrogate the structure on the size and shape of the defects, and to define the most appropriate order to follow for defect characterization. In particular, the simulations treated the problem of internal defects that could be difficult to study experimentally because of the practical inconvenience of making internal defects in a real plate. In the first three sections of the paper, we provide a statement of the problem and a description of the model used. Then, we present and discuss the results of the analyses for shear and Rayleigh–Lamb waves.

## 2. Guided Waves in Plates

**Figure 1:**
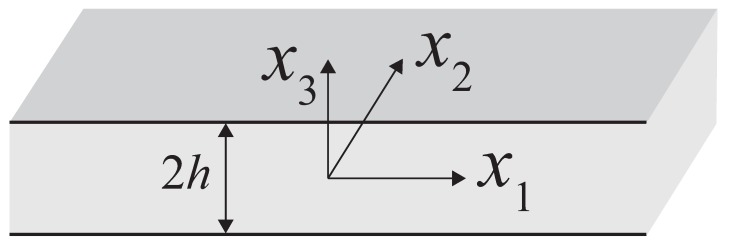
Plate.

In the absence of body forces, the vibrations of a three-dimensional homogeneous and isotropic elastic solid are described by the equation:

(1) $$div\sigma =\rho \ddot{u}$$

where $\ddot{u}$ is the second-order time derivative of the displacement vector; ρ is the material density; and σ=λtr(E)I+2μE is the stress tensor, with λ and μ as the Lamé constants, I as the identity tensor, and $E=(\nabla u+\nabla u^{T})/2$ as the strain tensor. In plates (Figure 1), bulk waves reflect between the free-stress boundaries, such that plane wavefront ($x_{2},\;x_{3}$) solutions travelling along the $x_{1},\;x_{3}$ plane exist [25]:

(2) $$u=Ue^{i[k\left(x_{1}+\alpha x_{3}\right)-\omega t]}$$

where *k* is the wavenumber along $x_{1}$, α is the ratio of the wavenumber in the $x_{3}$ direction to that along $x_{1}$, and ω is the angular frequency in rad/s. In such plane waves, no dependence on $x_{2}$ occurs. Substituting Equation (2) into Equation (1), the equations of motion decouple into two equations (first and third) involving displacements along $x_{1}$ and $x_{3}$, which are the Rayleigh–Lamb waves, and one equation (second) involving only displacements along $x_{2}$, which represents shear waves. The following eigenvalue problem in α is hence obtained:

(3) $$\left[\begin{array}{ccc} k^{2}\left[-\lambda -\left(2+\alpha^{2}\right)\mu \right]+\omega^{2}\rho & 0 & -\alpha (\lambda +\mu )\\ 0 & -k[\left(1+\alpha^{2}\right)\mu +\omega^{2}\rho & 0\\ -\alpha (\lambda +\mu ) & 0 & -k\left[\mu -\alpha^{2}\left(\lambda +2\mu \right)\right]+\omega^{2}\rho \end{array}\right]\left[\begin{array}{c} u_{1}\\ u_{2}\\ u_{3} \end{array}\right]=\left[\begin{array}{c} 0\\ 0\\ 0 \end{array}\right]$$

The solution of the characteristic equation deriving from Equation (3) provides six roots, which can be ordered in couples equal in modulus and opposite in sign. Four roots are related to Rayleigh–Lamb waves, and two are related to shear waves. Substituting these values in Equation (1), the displacement field is obtained, from which the stress field is derived through the constitutive equations. The free-stress boundary condition on $x_{3}=\pm h$ can now be set up, providing a second eigenvalue problem whose solution gives the values of *k* and enables determination of the dispersion relation.

**Figure 2:**
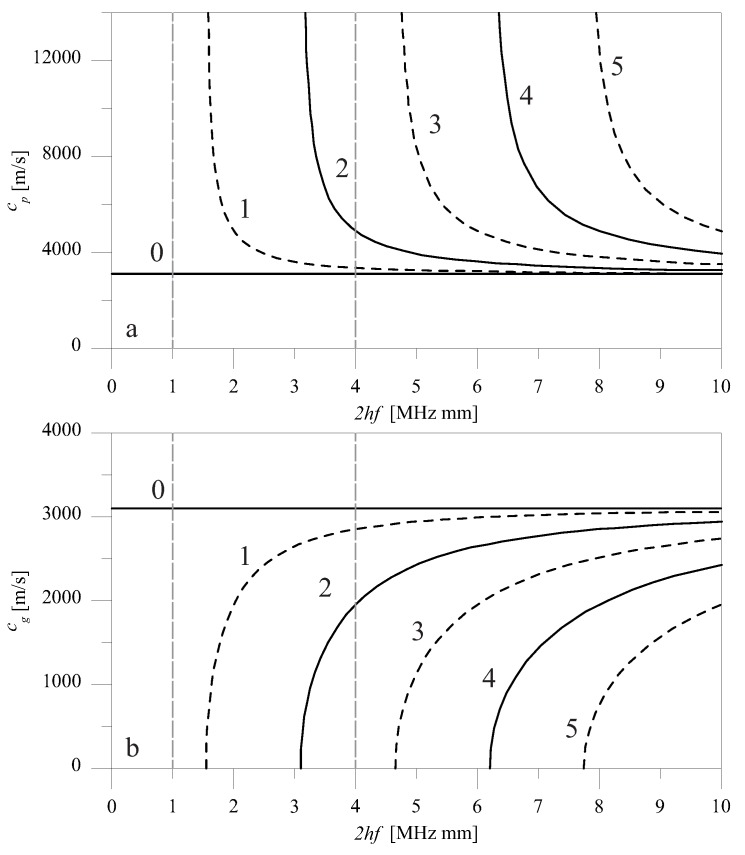
Phase (**a**) and group (**b**) velocity of shear waves in an aluminum plate.

**Figure 3:**
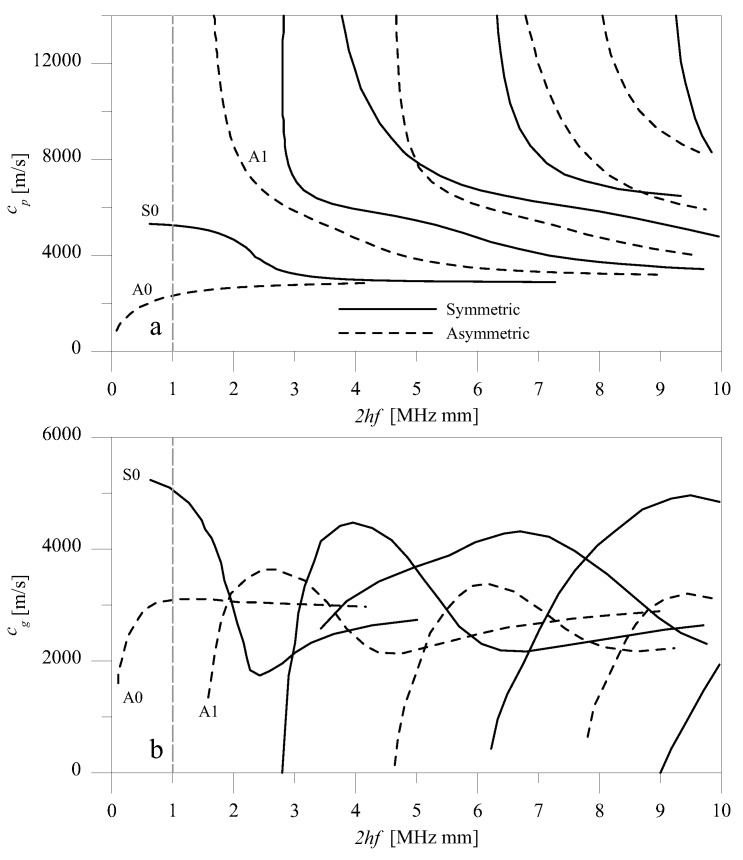
Phase (**a**) and group (**b**) velocity of Rayleigh–Lamb waves in an aluminum plate.

Figure 2 and Figure 3 respectively report the dispersion relation of shear and Rayleigh–Lamb waves, showing phase (a) and group velocity (b) as a function of the thickness-frequency product 2*hf* (in MHz mm), with frequency *f* (in Hz). The plot refers to the aluminum plate that was used in the examples, with the following parameters: ρ = 2810 kg/m${}^{3}$, μ = 27,000 MPa, and λ = 55,000 MPa. Longitudinal and shear velocities were respectively equal to $c_{L}$ = 6200 m/s and $c_{T}$ = 3071 m/s.

## 3. Interaction of Guided Waves with Discontinuities

When a guided wave meets geometric discontinuities, which can be flaws, voids, cavities or discontinuities of any kind, reflected and transmitted waves arise. Let us consider the simplest case, which is a single sharp change of cross-section, and assume that the incident wave is the *n*th wave mode with frequency ω in rad/s. We have one displacement component along $x_{2}$ for shear waves:

(4) $$u_{2n}^{inc}=U_{2n}\left(x_{3}\right)e^{i(k_{n}x_{1}-\omega t)}$$

and two components, along $x_{1}$ and $x_{3}$, for Rayleigh–Lamb waves:

(5) $$u_{1n}^{inc}=U_{1n}\left(x_{3}\right)e^{i(k_{n}x_{1}-\omega t)}\;u_{3n}^{inc}=U_{3n}\left(x_{3}\right)e^{i(k_{n}x_{1}-\omega t)}$$

where $U_{n}\left(x_{3}\right)$ is the *n*th wave mode shape, and $k_{n}$ is its wavenumber.

The far-field harmonic response $u^{l}$ at a point before the discontinuity can be expressed as the superposition of the incident wave mode plus a reflected wave field, represented by the sum of wave modes with reflection coefficients $R_{np}$. The subscripts *n* and *p* specify both the *n*th incident and *p*th reflected wave modes. The response can hence be written as:

(6) $$u_{2}^{l}=u_{2n}^{inc}+\sum_{p=0}^{N}R_{np}^{S}U_{2p}\left(x_{3}\right)e^{i(-k_{p}x_{1}-\omega t)}$$

for shear waves, and

(7) $$u_{1}^{l}=u_{1n}^{inc}+\sum_{p=0}^{N}R_{np}^{RL}U_{1p}\left(x_{3}\right)e^{i(-k_{p}x_{1}-\omega t)}\;u_{3}^{l}=u_{3n}^{inc}+\sum_{p=0}^{N}R_{np}^{RL}U_{3p}\left(x_{3}\right)e^{i(-k_{p}x_{1}-\omega t)}$$

for the two components of the Rayleigh–Lamb waves. The summation index extends to the *N* modes propagating at the given frequency. Regarding the transmitted wave field $u^{r}$, the superposition of the *N* wave modes is expressed as a summation of waves with transmission coefficients $T_{np}$, that is:

(8) $$u_{2}^{r}=\sum_{p=0}^{N}T_{np}^{S}U_{2p}^{r}\left(x_{3}\right)e^{i(k_{p}^{r}x_{1}-\omega t)}$$

for shear waves, and

(9) $$u_{1}^{r}=\sum_{p=0}^{N}T_{np}^{RL}U_{1p}^{r}\left(x_{3}\right)e^{i(k_{p}^{r}x_{1}-\omega t)}\;u_{3}^{r}=\sum_{p=0}^{N}T_{np}^{RL}U_{3p}^{r}\left(x_{3}\right)e^{i(k_{p}^{r}x_{1}-\omega t)}$$

for the two components of Rayleigh–Lamb waves. The superscript *r* in the wavenumbers and wave modes of Equations (8) and (9) indicates that they depend on the mechanical and geometrical characteristics of the right part of the waveguide. The index *N* in Equations (6)–(9) can, in principle, be different. In the absence of energy loss, the sum of the power flow of the incident wave must be equal to the sum of the energy flows of the reflected and transmitted waves.

The coefficients $R_{np}$ and $T_{np}$ can be calculated analytically by making use of the principle of reciprocity in elastodynamics, with a method presented in [22]. In short, a relation between two states, in the presence and in the absence of the discontinuity, was established and projected onto the wave basis of the undamaged structure. The requirement that the reciprocity condition is satisfied restores congruence and balance, and is a practical way to set up boundary conditions in integral form at a discontinuity. This approach is limited by the fact that it can be applied only to sharp changes in height, be they symmetric or nonsymmetric, as the wave modes are only defined onto a waveguide of constant height or cross-section. If the discontinuity is of notch-type, which can be described by a double change of height, the mentioned relations are established twice, that is, one time for each change in height.

**Figure 4:**
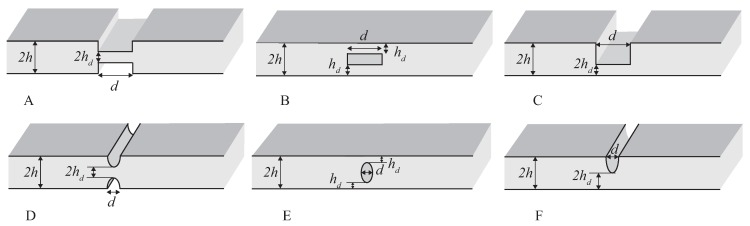
Profiles of discontinuities and voids under investigation: external symmetric (**A**,**D**); internal symmetric (**B**,**E**); external nonsymmetric (**C**,**F**).

The derived coefficients $R_{np}$ and $T_{np}$ depend on the geometry of the discontinuity, which, in the case of a single sharp change of cross-section, is described by the ratio between the depth of the notch $h-h_{d}$ and the undamaged height *h*, with residual height $h_{d}$, that is, $r=(h-h_{d})/h$, and by the ratio $\delta =d/\lambda_{w}$, where *d* is the length of the notch and $\lambda_{w}$ is the wavelength of the incident wave (Figure 4A). The parameter *r* measures the notch magnitude, as r=0 corresponds to a continuous plate, whereas r=1 corresponds to a fully cracked cross section. To clarify such dependence, we have investigated how different kinds of waves interact with voids of different shapes, as depicted in Figure 4. A double sharp discontinuity (Figure 4A–C) and an elliptic profile (Figure 4D–F) were studied considering different symmetries: external symmetric (A,D), internal symmetric (B,E), and external nonsymmetric (C,F). The nondimensional parameters used to describe such voids were again the ratio *r* and δ. Different sizes of the discontinuity were considered by varying *r* and δ. To study the scattering for these profiles, all the cases were studied with an FE model, using the analytical model as a reference.

## 4. Finite Element Model of Guided Waves in a Plate

**Figure 5:**
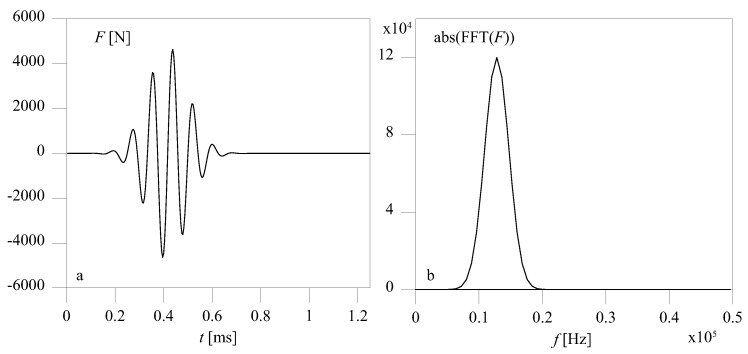
Time-history (**a**) and Fourier transform (**b**) of the forcing function.

**Figure 6:**
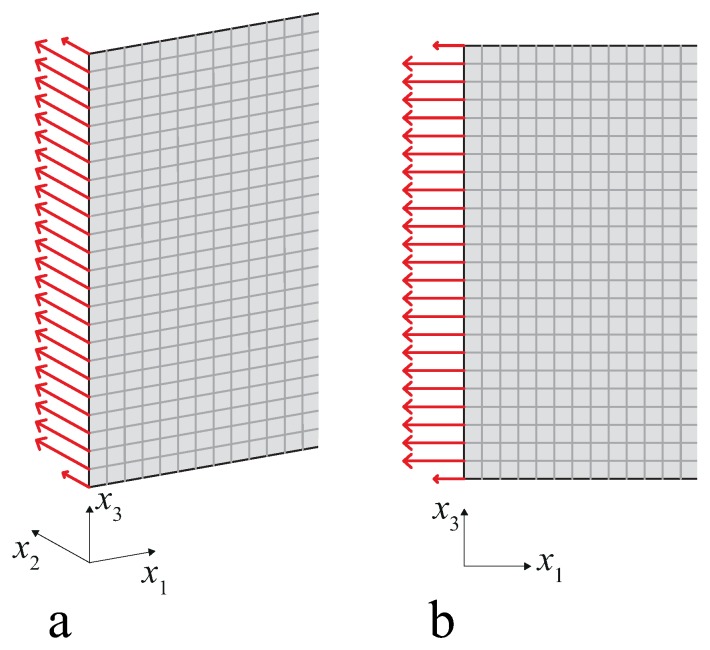
Spatial distribution of forces used to generate SH0 (**a**) and S0 (**b**) waves.

Two plane models were developed: a state of plane strain with in-plane displacements describing Rayleigh–Lamb waves, and an axisymmetric model with a very large radius and out-of-plane displacements, which describes shear waves. In fact, the shear solution tends to a torsional solution for large radii. The different kinds of waves investigated were generated by exciting one free end of the plate with a sine burst that we obtained as a sine wave modulated with a Gaussian window including around six periods. The time-history and Fourier transform of the forcing function are reported respectively in Figure 5a,b. The sine burst was used to select a narrow frequency band and reduce dispersion phenomena, and had an appropriate spatial distribution so that different incident waves could be modeled, as shown in Figure 6. This distribution of forces was merely a finite element strategy and is highly impracticable for laboratory tests. In practice, guided waves are generated with piezoelectric patches glued on the surface of the plate, whose strain time-history is electrically driven [26]. By using two in-phase transducers on the two surfaces of the plate, which apply displacements in the plane of the plate, an S0 wave can be easily generated. In an isotropic plate, the SH0 wave results as a by-product of this excitation at an angle of π/4 with respect to the direction of the displacement applied.

The spatial distribution of forces of Figure 6a,b generate, respectively, SH0 and S0 waves. For shear waves, two regimes of the product 2*hf* were investigated, which we will call low- (2*hf* = 1 MHz mm) and high- (2*hf* = 4 MHz mm) frequency height regimes (Figure 2). These different regimes were modeled by modifying the thickness of the plate. They differ in the fact that when 2*hf* = 1 MHz mm, the only propagating modes are SH0 for shear waves, but when 2*hf* = 4 MHz mm, SH1 and SH2 are also present. For Rayleigh–Lamb waves, only the low-frequency regime with 2*hf* = 1 MHz mm was considered, where both S0 and A0 propagate (Figure 3).

This dynamic problem was solved with a transient analysis by the Newmark time integration method. Time and space resolutions were chosen so that accurate results were obtained. In particular, the time step used was 1/20 of the frequency, and the maximum size of the elements was 1/20 of the maximum wavelength involved. Structural damping was neglected, and a linear elastic material was used, with the mechanical properties listed in Section 2. The depths of the discontinuities that were investigated corresponded to five values of *r* = 0.17, 0.33, 0.50, 0.67, and 0.83.

## 5. Interaction of the Shear (SH0) Mode with Discontinuities

**Figure 7:**
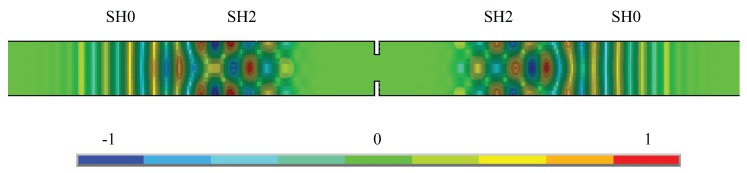
Contour plot of $u_{2}$ displacements at a time step following the interaction of the SH0 mode with an A-type discontinuity (2*hf* = 4 MHz mm).

A qualitative description of the interaction between the SH0 mode and an A-type discontinuity (Figure 5) is provided in Figure 7, which reports the contour plot of displacements $u_{2}$ at a time instant after the SH0 wave had encountered the discontinuity. The case depicted in Figure 7 concerned a 2*hf* regime in which SH0, SH1 and SH2 waves could, in principle, propagate, and shows that the scattered response contained only the SH0 and SH2 modes. This result could be explained by observing that the contribution of nonsymmetric modes vanishes if the discontinuity is symmetric when one sets up the boundary conditions in the analytical form obtainable from the principle of reciprocity.

The coefficients of reflection and transmission were extracted from the FE results by observing the amplitude of the Fourier transforms of the deformed shape along a straight line parallel to the axis at an appropriately chosen time. To operate in this way, the function $\exp^{-ikx}$ must be assumed to be the kernel of the Fourier transform, establishing a duality between space and wavenumber instead of the usual time frequency. The line must be chosen appropriately for each mode, that is, where the amplitude of its mode shape is at a maximum. In such a way, the peak observed in the Fourier transform gives the amplitude of the wave mode that is afterwards normalized by the amplitude of the incident mode. We considered first the low- (2*hf* = 1 MHz mm) frequency height regime, and then the high- (2*hf* = 4 MHz mm) frequency height regime.

### 5.1. Low *2*hf Regime

**Figure 8:**
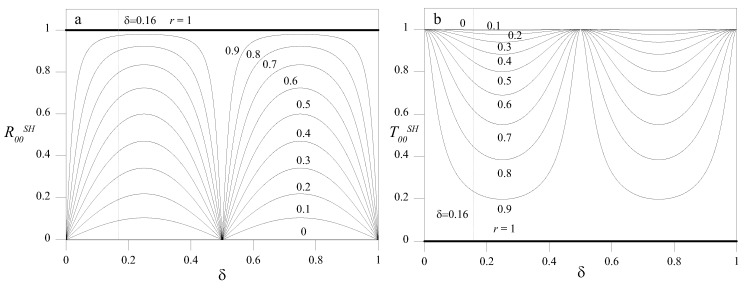
$R_{00}^{SH}$ (**a**) and $T_{00}^{SH}$ (**b**) for several values of *r* as a function of $\delta =d/\lambda_{w}$ (2*hf* = 1 MHz mm).

In the low- (2*hf* = 1 MHz mm) frequency height regime, only SH0 wave propagated in the far-field (Figure 2). Hence, when SH0 wave encountered a discontinuity, whatever its shape, the only propagating mode retrieved in the scattered response was the SH0 mode. As a first step, we will elucidate how $R^{SH}$ and $T^{SH}$ varied for a double sharp change of cross section. This situation is a case that can be easily solved analytically by exploiting the principle of reciprocity in elastodynamics. Figure 8a,b shows the analytical variation of $R_{00}^{SH}$ and $T_{00}^{SH}$ as a function of δ for several values of *r* for an A-type discontinuity. A periodic pattern is shown, with maxima and minima of both reflection and transmission coefficients occurring at integer multiples of the ratio δ=n/2. Such maxima and minima were due to the constructive or destructive interference of the reflection between the two changes of cross-section [10], and were also retrieved experimentally in [27]. It is interesting to observe that such properties of the double sharp change of cross-section made this case very similar to the Fabry-Pérot interferometer in optics, which was used to measure the wavelength of light. Conversely, in the context of guided waves, this could be exploited to measure the extension of the discontinuity.

**Figure 9:**
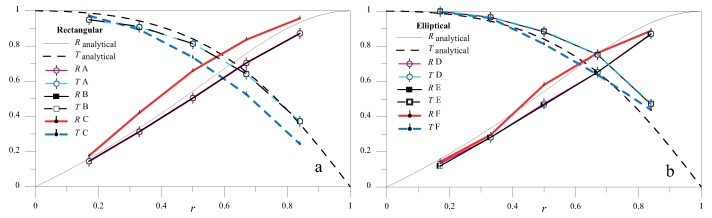
$R_{00}^{SH}$ and $T_{00}^{SH}$ as a function of *r* for δ=0.16 for rectangular (**a**) and elliptical (**b**) profiles of the notch.

For δ=0.16 (Figure 8), the dependence of $R^{SH}$ and $T^{SH}$ on *r* is shown in Figure 9a for double sharp changes of the cross-section and in Figure 9b for elliptical profiles. Apart from the continuous lines, both solid and dashed, that refer to the analytical solution obtained for case A, all the discrete results were obtained from an FE model. All the cases examined have a trend that closely resembles that of the analytical results. The response was exactly the same for cases A and B, and for cases D and E. This result means that, provided that the notch is symmetric, for such values of 2*hf*, the response is insensitive to the in-depth location of the notch. Moreover, the difference between the couples A–B and D–E are extremely limited, which indicates a feeble dependence of the response on the profile of the notch. The nonsymmetric cases C and F present some deviations from the other cases. This result was because, in order to satisfy the boundary conditions, local, non-propagating, nonsymmetric modes arose.

### 5.2. High *2*hf Regime

**Figure 10:**
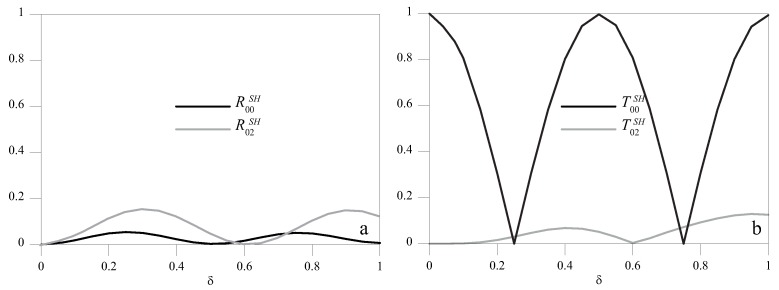
$R_{00}^{SH}$ and $R_{02}^{SH}$ (**a**) and $T_{00}^{SH}$ and $T_{02}^{SH}$ (**b**) as a function of δ for r=0.1.

In the high- (2*hf* = 4 MHz mm) frequency height regime, SH0, SH1 and SH2 waves could, in principle, propagate (Figure 2). For an A-type discontinuity, the dependence on δ obtained from the analytical model of $R_{00}^{SH}$, $R_{02}^{SH}$, $T_{00}^{SH}$ and $T_{02}^{SH}$ for r=0.1 is presented in Figure 10. Similarly to that occurring at low frequencies, we observed a periodic pattern in all the scattered components. The normalized spatial period, which was n/2 for the SH0 mode, is longer for the SH2 mode, as this has a larger wavelength.

**Figure 11:**
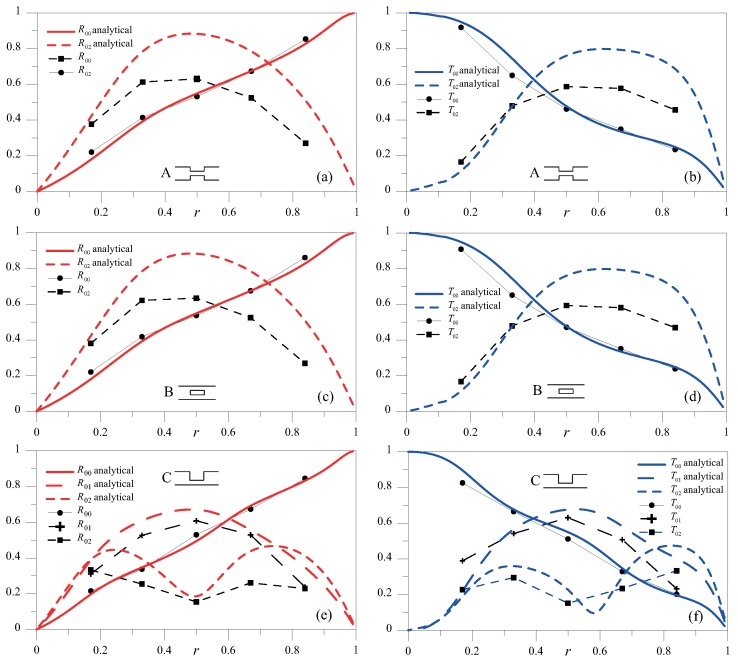
$R_{00}^{SH}$, $R_{01}^{SH}$ and $R_{02}^{SH}$ (**a**,**c**,**e**) and $T_{00}^{SH}$, $T_{01}^{SH}$ and $T_{02}^{SH}$ (**b**,**d**,**f**) as a function of *r* for δ=0.16 for cases A–C.

**Figure 12:**
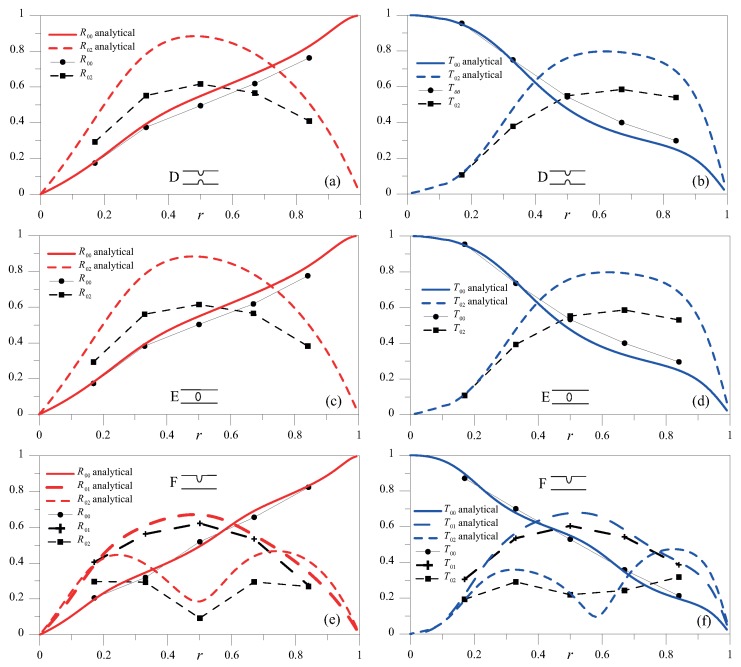
$R_{00}^{SH}$, $R_{01}^{SH}$ and $R_{02}^{SH}$ (**a**,**c**,**e**) and $T_{00}^{SH}$, $T_{01}^{SH}$ and $T_{02}^{SH}$ (**b**,**d**,**f**) as a function of *r* for δ=0.16 for cases D–F.

For δ=0.16, with the SH0 mode incident, Figure 11 and Figure 12 show the coefficients of reflection and transmission of the three modes involved for all the discontinuity shapes under investigation, together with the analytical solution obtained using the principle of reciprocity for the A-type discontinuity. It can be observed that, when SH0 wave encountered a symmetric discontinuity (cases A–E), only the SH0 and SH2 modes were found in the scattered response, whereas if the discontinuity was asymmetric (cases C and F), the SH1 mode also emerged. Similarly to that occurring for 2hf = 1 MHz mm, the numerical results closely resembled the analytical results, and, more importantly, the scattered field had the same amplitude for coupled cases A–B and D–E; that is, it did not depend on the notch profile.

## 6. Interaction of Rayleigh–Lamb Wave Mode S0 with Discontinuities

**Figure 13:**
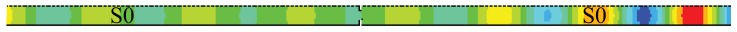
Contour plot of $u_{1}$ displacements at a time step following the interaction of the S0 mode with an A-type discontinuity (2hf = 1 MHz mm).

**Figure 14:**
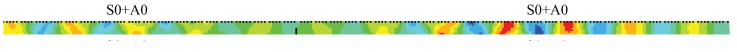
Contour plot of $u_{1}$ displacements at a time step following the interaction of the S0 mode with a C-type discontinuity (2hf = 1 MHz mm).

For Rayleigh-Lamb modes, the analysis was limited to the low- (2hf=1 MHz mm) frequency height regime, which already contains a symmetric S0 and an asymmetric A0 mode. Figure 13 reports an FE qualitative description of the interaction of the S0 mode with a symmetric A-type discontinuity. This figure reports a contour plot of $u_{1}$ displacements at a time step following the interaction between the incident mode and the discontinuity. It shows that when S0 waves encountered an A-type symmetric discontinuity, the scattered response contained only the S0 mode, as the A0 mode was not required to satisfy the boundary conditions. In contrast, when S0 waves encountered a nonsymmetric C-type discontinuity, the response contained both the incident S0 and the nonsymmetric A0 mode, which was then necessary to satisfy the boundary conditions. The evidence for this statement is given in Figure 14, which shows the displacement field $u_{1}$ at a time step after the S0 mode had encountered a nonsymmetric discontinuity. The interaction of the S0 mode with symmetric discontinuities is presented first, followed by the interaction with nonsymmetric discontinuities.

**Figure 15:**
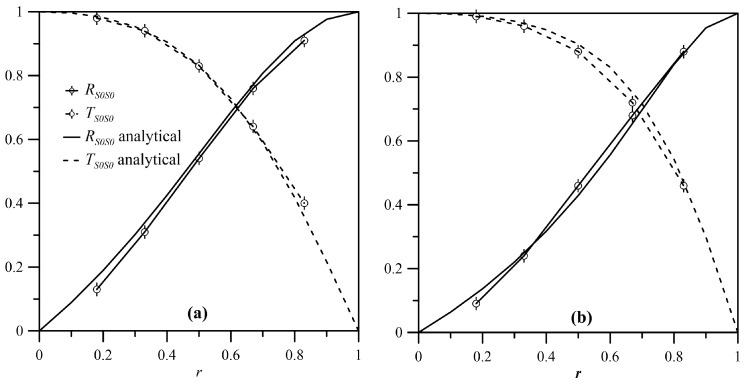
$R_{S_{0}S_{0}}^{RL}$ and $T_{S_{0}S_{0}}^{RL}$ for δ = 0.13 (**a**) and δ = 0.07 (**b**) for the A-type discontinuity.

When the S0 mode interacted with a symmetric discontinuity, the reflection and transmission coefficients exhibited a dependence on δ that was similar to that obtained for shear waves, as reported in Figure 8. Figure 15 reports the reflection and transmission coefficients for a symmetric A-type discontinuity as a function of *r* for two different values of δ=0.13 (a) and δ=0.07 (b). The curves present a pattern analogous to that of the SH0 mode (see Figure 9), and show that there was a remarkable similarity between the analytical and FE model results.

**Figure 16:**
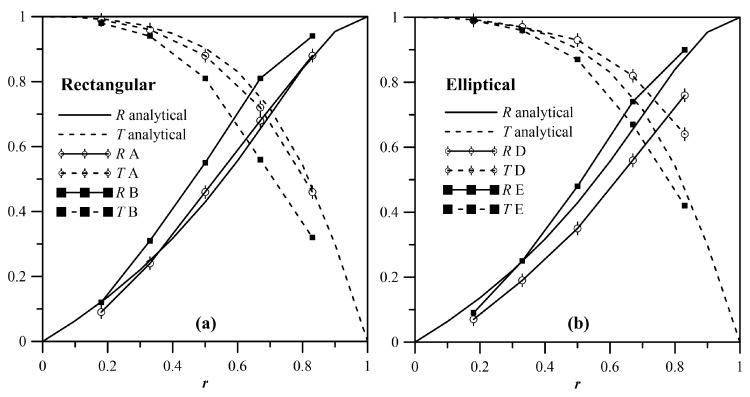
$R_{S_{0}S_{0}}^{RL}$ and $T_{S_{0}S_{0}}^{RL}$ for rectangular (**a**) and elliptical voids (**b**); δ=0.07.

Given δ=0.07, Figure 16 reports the dependence of $R_{S_{0}S_{0}}^{RL}$ and $T_{S_{0}S_{0}}^{RL}$ on *r* for the sharp changes of the cross-section cases A and B (a), and for the elliptical profile cases D and E (b). The case that was best described by the analytical results is clearly case A, whereas the others presented some deviation. Differently to the case concerning shear waves, here, cases A and B, and D and E, differed from each other. This result was due to the in-plane Poisson effect, an effect which is tied to the notch profile. Regardless, the differences between the different cases were limited, which indicated a feeble dependence of the response on the profile of the notch.

**Figure 17:**
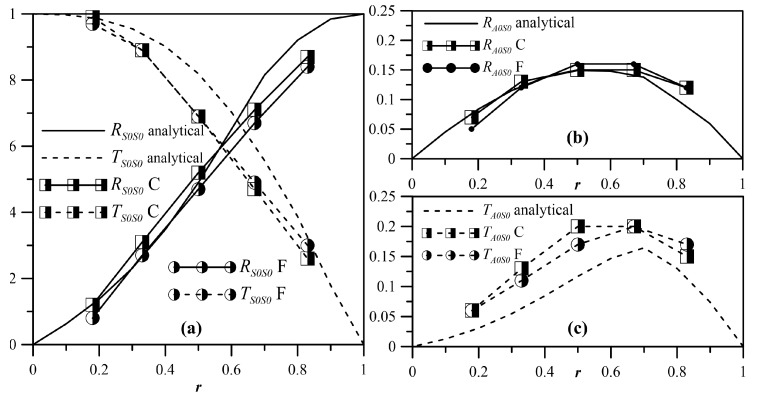
$R_{S_{0}S_{0}}^{RL}$ and $T_{S_{0}S_{0}}^{RL}$ (**a**), $R_{S_{0}A_{0}}^{RL}$ (**b**) and $T_{S_{0}A_{0}}^{RL}$ (**c**) for cases C and F; δ=0.07.

Figure 17, again for δ=0.07, shows the coefficients $R_{S_{0}S_{0}}^{RL}$, $R_{S_{0}A_{0}}^{RL}$, $T_{S_{0}S_{0}}^{RL}$ and $T_{S_{0}A_{0}}^{RL}$ as a function of *r* for the asymmetric cases C and D. Differently to that occurring for symmetric discontinuities, here, we also had the contribution of asymmetric modes, that was null when *r* = 0 or *r* = 1, and reached a maximum for middle values of the damage intensity. This result was again because, for middle values of *r*, the contribution of asymmetric modes was needed to satisfy the boundary conditions. The agreement between the numerical and analytical results was reasonably good.

## 7. Conclusions

We described the interaction of SH0 (shear) and S0 (Rayleigh–Lamb) wave modes with a discontinuity such as a notch or an internal void in a plate. A finite element model was used to model different profiles, whereas an analytical solution obtainable for the double sharp change of the cross-section was maintained as a reference and exploited to elucidate some details of the interaction. Reflection and transmission coefficients of the scattered field were evaluated considering the different sizes and profiles of the discontinuity, such as step changes in height or elliptical shapes (internal or external and symmetric or nonsymmetric).

It was shown that, depending on the value of the product 2hf, the far-field scattered signal depends on the number of wave modes that propagate at that given frequency, as indicated by the dispersion relation. For low values of the product 2hf, for which shear waves support only one propagating mode, when the SH0 mode is incident, only that single mode will be contained in the far-field response, irrespective of the shape and symmetry of the discontinuity. Other modes necessary to satisfy the boundary conditions were only retrieved locally. Differently to shear modes, Rayleigh–Lamb modes supported at least symmetric S0 and nonsymmetric A0 modes for low values of the product 2hf. When several propagating modes exist and an incident symmetric mode (SH0 or S0) encounters a symmetric discontinuity, only symmetric modes will exist in the scattered response. If the discontinuity is nonsymmetric, both symmetric and nonsymmetric modes will exist. This result is probably the most useful for applications, as it could be used to discern symmetric from nonsymmetric discontinuities. It was also found that the dependence of the *R* and *T* coefficients on the profile of the discontinuity was limited, which made it difficult to detect the shape of the profile in the presence of experimental errors. Further confirming the previous results of the authors, it was also shown that *R* and *T* depended both on the depth and on the extension of the discontinuity. Moreover, in the presence of several propagating modes, the contribution of modes other than the incident mode was more important for mid-range values of the ratio *r*, where these modes contributed to satisfying the boundary conditions.
